# Nuclear distribution and chromatin association of DNA polymerase α-primase is affected by TEV protease cleavage of Cdc23 (Mcm10) in fission yeast

**DOI:** 10.1186/1471-2199-6-13

**Published:** 2005-06-07

**Authors:** Xiaowen Yang, Juraj Gregan, Karola Lindner, Hedi Young, Stephen E Kearsey

**Affiliations:** 1 Department of Zoology, University of Oxford, South Parks Road, Oxford OX13PS UK; 2 Current address: Structural Genomics Consortium, Nuffield Department of Clinical Medicine, Botnar Research Centre, University of Oxford, Oxford OX3 7LD, UK; 3 Current address: IMP, Dr. Bohr-Gasse 7, A-1030, Austria

## Abstract

**Background:**

Cdc23/Mcm10 is required for the initiation and elongation steps of DNA replication but its biochemical function is unclear. Here, we probe its function using a novel approach in fission yeast, involving Cdc23 cleavage by the TEV protease.

**Results:**

Insertion of a TEV protease cleavage site into Cdc23 allows in vivo removal of the C-terminal 170 aa of the protein by TEV protease induction, resulting in an S phase arrest. This C-terminal fragment of Cdc23 is not retained in the nucleus after cleavage, showing that it lacks a nuclear localization signal and ability to bind to chromatin. Using an in situ chromatin binding procedure we have determined how the S phase chromatin association of DNA polymerase α-primase and the GINS (Sld5-Psf1-Psf2-Psf3) complex is affected by Cdc23 inactivation. The chromatin binding and sub-nuclear distribution of DNA primase catalytic subunit (Spp1) is affected by Cdc23 cleavage and also by inactivation of Cdc23 using a degron allele, implying that DNA polymerase α-primase function is dependent on Cdc23. In contrast to the effect on Spp1, the chromatin association of the Psf2 subunit of the GINS complex is not affected by Cdc23 inactivation.

**Conclusion:**

An important function of Cdc23 in the elongation step of DNA replication may be to assist in the docking of DNA polymerase α-primase to chromatin.

## Background

Proteases play key roles in the regulation of many cellular processes, such as signal transduction, apoptosis, and the activation of chromosome disjunction in mitosis [1-4]. Artificial proteolytic regulation is possible using TEV protease, which recognizes a highly specific sequence [5] and is not deleterious when expressed in a variety of cell types [6-11]. Artificial cleavage of target proteins, engineered to contain a TEV cleavage sequence (Tcs), can thus be effected by TEV protease expression in vivo. TEV protease-mediated cleavage has been used in topological studies of protein location [12] and to study the role of regulatory proteolysis, such as in analysis of separase function [9]. Proteolytic cleavage can be used to determine how removal of specific domains of a protein affects its function [13] and, by removing a signal that targets the protein to a specific compartment, can effect a change in a protein's cellular localization [11].

We investigate here the utility of TEV-mediated cleavage of nuclear proteins in fission yeast. We demonstrate that a strain expressing nuclear-targeted TEV protease has no growth defects compared to a wild-type strain and shows efficient cleavage of nuclear proteins containing a Tcs. We use TEV protease-mediated cleavage to investigate the function of Cdc23 (homologous to Mcm10 in other organisms), which is an essential replication protein, required for the initiation and elongation steps of DNA replication ([14-17], see [18,19] for reviews). Cdc23/Mcm10 is chromatin associated, and in *S. cerevisiae *and vertebrate cells this association occurs during S phase [20-22], but its biochemical function is unclear. In *S. cerevisiae*, Mcm10 was originally implicated in pre-replicative complex formation, when Mcm2-7 proteins associate with ORC at replication origins [23]. Subsequent studies in both yeasts and *Xenopus *have shown that Mcm10 is required for the later step of replication initiation, as the protein is needed for chromatin association of Cdc45 [21,24,25]. In vitro, fission yeast Cdc23 enhances the ability of Hsk1 (Cdc7) protein kinase to phosphorylate Mcm2 [26], and binds to the catalytic subunit of DNA polymerase α-primase (pol α-primase), catalytically activating its DNA polymerase activity [27]. Very recently, Ricke and Bielinsky have shown that Mcm10 is required for the stability of the pol α catalytic subunit in *S. cerevisiae *[20].

In this paper, we use TEV protease cleavage of Cdc23 to show that removal of a 170 aa C-terminal domain of the protein, previously with no attributed functions, blocks DNA replication. Regulation of TEV protease under the thiamine-repressible *nmt1 *promoter in conjunction with a Tcs-containing allele of *cdc23 *can thus be used to inactivate conditionally the protein. We show in addition that inactivation of Cdc23 affects the chromatin binding and nuclear distribution of the DNA primase catalytic subunit (Spp1) of pol α-primase.

## Results

### Expression of TEV protease in *S. pombe *and cleavage of Mcm7-Tcs-GFP

**Figure 1:**
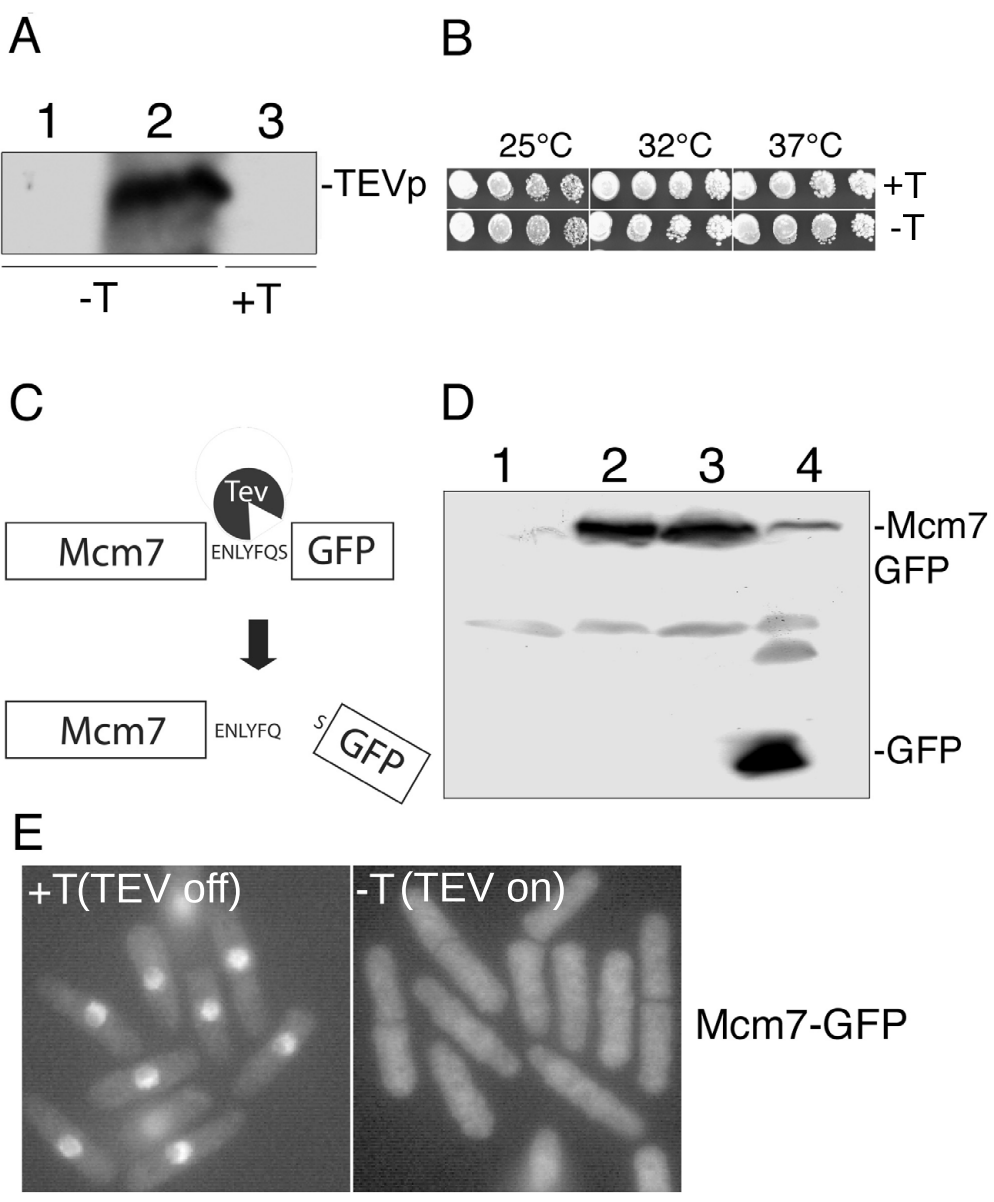
**Effects of TEV protease expression on wild-type and *mcm7-GFP_N760_::Tcs S. pombe *strains**. A. Western analysis comparing levels of expression of TEV protease in strain P1292 in – thiamine medium (lane 2) and + thiamine medium (lane 3). Lane 1 is protein extract from a strain lacking the pREP3X-TEV-NLS plasmid (P138). B. Expression of TEV protease does not affect the viability of *S. pombe*. Serial dilutions of strain P1292 were spotted onto plus (+T) and minus (-T) medium and incubated at the temperatures shown. C. Design of Mcm7-GFP_N760_::Tcs. The Mcm7 gene was modified so that a TEV cleavage site, fused to GFP is expressed after the last codon of the Mcm7 reading frame. Cleavage by TEV protease liberates GFP with an N- terminal serine, which is stable according to the N-end rule. D. Western analysis of the *mcm7-GFP*_*N760*_*::Tcs *strains, using anti-GFP antibody. Lane 1: untagged control strain (P138); lane 2: *mcm7-GFP*_*N760*_*::Tcs *strain (P1288); lane 3: *mcm7-GFP*_*N760*_*::Tcs *containing pREP3X-TEV-NLS, +thiamine (P1171); lane 4: *mcm7-GFP*_*N760*_*::Tcs *strain containing pREP3X-TEV-NLS, -thiamine (P1171). All cultures were grown at 32°C. D. Fluorescence microscopy analysis of cells expressing Mcm7-GFP_N760_::Tcs in the absence (+thiamine) or presence (-thiamine) of TEV protease. Cells were fixed using methanol and acetone.

To explore the utility of using TEV protease to construct conditional alleles in *S. pombe*, we constructed a strain where TEV protease, conjugated to nuclear localization signals (NLSs), is expressed under the control of the thiamine-regulatable *nmt1 *promoter. A strain expressing TEV protease is viable over a range of temperatures (25–37°C, Fig. 1A, B), and no effects on growth rate or DNA contents of cells as assessed by flow cytometry were detected (data not shown). To demonstrate that this TEV protease is capable of cleaving nuclear proteins, we modified a strain expressing Mcm7-GFP to contain a Tcs between the C-terminus of Mcm7 and GFP (Mcm7-GFP_N760_::Tcs). Mcm7 forms a complex with five other Mcm subunits (Mcm2-6), and probably provides the helicase function during the initiation and elongation steps of DNA replication (reviewed in [28,29]). Western blot analysis showed that expression of TEV protease efficiently removed the GFP tag from Mcm7 (Fig. 1D). Mcm7 is present in the nucleus throughout the cell cycle [14] (Fig. 1E, +T), in common with other Mcm subunits, but on expression of the TEV protease, the GFP fluorescence becomes delocalized over the entire cell (Fig. 1E), showing that the test substrate is efficiently cleaved on protease induction. The small size of the C-terminal GFP fragment presumably allows it to exit the nucleus by passive diffusion, consistent with earlier studies[30].

### Inactivation of Cdc23 by TEV protease cleavage

**Figure 2:**
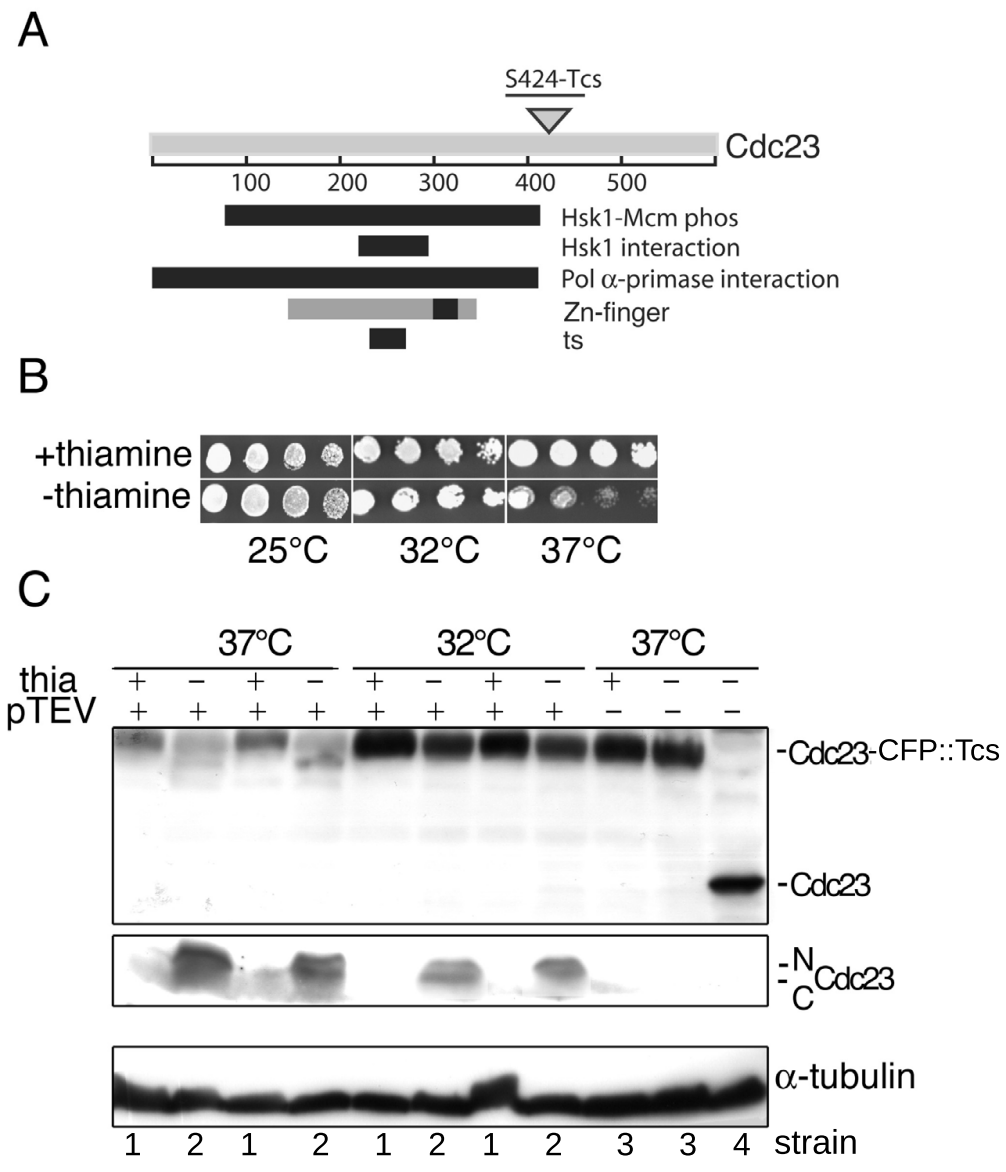
**Construction and characterization of a strain expressing Cdc23-CFP with an internal TEV cleavage site (Cdc23_S424_::Tcs)**. A. Regions of Cdc23 with attributed functions (see text for details), and location of Tcs in Cdc23_S424_::Tcs. The grey bar superimposed on the black Zn-finger region corresponds to the domain of *S. cerevisiae *Mcm10 required for homocomplex assembly [31]. "ts" indicates the location of *cdc23-1E2 and -M36 *ts mutations. B. Expression of TEV protease causes loss of viability of a *cdc23*_*S424*_*::Tcs *strain (P1323) at 37°C. Serial dilutions of the P1323 strain were spotted onto minus or plus thiamine media and incubated at the temperatures shown. C. Western analysis of Cdc23 levels in *cdc23*_*S424*_*::Tcs *strains in presence or absence of pTEV (pREP3X-TEV-NLS) plasmid, grown in the presence or absence of thiamine. The blot was probed with antibodies against Cdc23, and α-tubulin is shown as a loading control. Cells were first grown at 25°C in the presence of thiamine to repress TEV protease expression, then washed and grown either in the presence or absence of thiamine for 24 h at the indicated temperatures, before extracting protein for western analysis. Cdc23_S424_::Tcs indicates the position of full length protein, N & C Cdc23 indicate the fragments (49 kDa (N) and 46 kDa (C)) after cleavage by TEV protease. Comparison of Cdc23 levels in strains 3 and 4 (wt) shows that the modification of Cdc23 does not affect protein levels under conditions where TEV protease is absent. Strains used were: (1) P1323-1; (2) P1323-2; (3); P1322 (4) P138.

A number of functions have been ascribed to the Cdc23/Mcm10 replication factor and some of these have been mapped to specific domains of the protein (Fig. 2A), such as in vitro stimulation of Hsk1 phosphorylation of Mcm2 [26] and stimulation of pol α-primase function [27]. Mcm10 contains a region encompassing a Zn-finger motif that is important for formation of high molecular weight Mcm10 homocomplexes [31] and a large segment of the protein has been reported to be needed for interaction with the Mcm2-7 complex. Most of these domains map to the N-terminal and central regions of the protein (corresponding to aa ca. 1–400 for the *S. pombe *protein, Fig 2A). All mapped *cdc23 *temperature-sensitive (ts) mutations also map to a central (232-269 aa) region. The function of the C-terminal portion of the protein, which is less well conserved than N-terminal and central domains, is unclear as a C-terminally truncated version of *S. pombe *Cdc23 (aa 1-423) can complement the *cdc23-M36 *mutant [26]. However, the C-terminal region of *S. cerevisiae *Mcm10 contains two NLSs, at least one of which is needed for function of the protein [32].

To determine if removal of a C-terminal domain of the Cdc23 replication factor affects the function of the protein, we inserted a Tcs just after serine 424 of a CFP-tagged version of Cdc23, 170 aa from the C terminus, thus generating Cdc23_S424_::Tcs, (Fig 2A). Amino acids 1-424 of Cdc23 contains most domains with ascribed functions and it was of interest to determine how cleavage after this region would affect the function of the protein. A poorly conserved region was chosen for the Tcs insertion to minimize the possibility that this modification alone would inactivate the protein. The expression level of Cdc23_S424_::Tcs is the same as the wild-type protein (Fig. 2C). A strain containing this insertion is viable at all temperatures in the absence of TEV protease, implying that the Tcs insertion does not affect Cdc23 function, but induction of the TEV protease prevents growth of this strain (Fig. 2B). Unexpectedly, this effect shows temperature-sensitivity, in that the strain grows at temperatures up to about 32°C, but is not viable at 37°C. The cleavage site used generates a C-terminal fragment with a stabilizing N-terminal amino acid (serine) as predicted by the N-end rule [33], and we could detect two fragments of the expected size after induction of the protease (Fig. 2C). The proportion of full-length protein is reduced at 37°C on TEV protease expression, consistent with the temperature-sensitivity of the strain. The effect is unlikely to be due to an increase in TEV protease activity, since the enzyme is more active at 32°C than 37°C [34] and we observed efficient cleavage of a different substrate (Mcm7-GFP_N760_::Tcs) at 32°C (Fig. 1). Instead, the Tcs in Cdc23 is presumably more accessible to the protease at higher temperatures, perhaps due to a change in conformation of Cdc23, allowing more efficient cleavage to occur.

**Figure 3:**
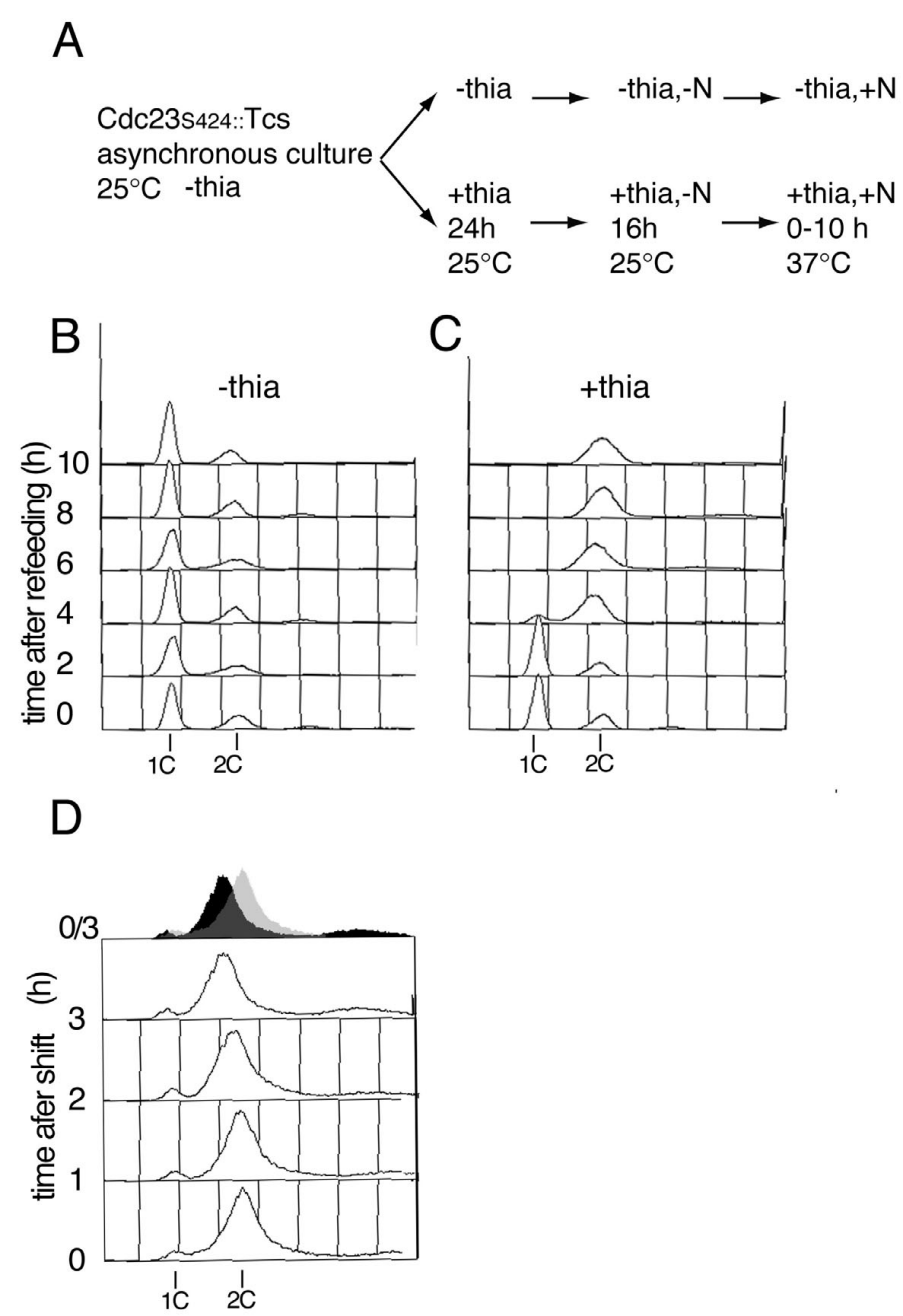
**Flow cytometry analysis of *cdc23_S424_::Tcs *strains following induction of TEV protease**. A. Scheme for experiment shown in (B) & (C). B-C. Flow cytometric analysis of G1-arrested *cdc23_S424_::Tcs *strain (P1376), released from a G1 block in the absence (B) or presence (C) of thiamine, showing arrest of DNA replication when TEV protease is expressed. D. Flow cytometric analysis of *cdc23_S424_::Tcs *strain (P1376) expressing TEV protease (i.e. – thiamine). The strain was grown to log phase at 25°C in – thiamine medium and shifted to 37°C at t = 0. The top panel superimposes the t = 0 (grey) and t = 3 h (black) histograms.

Cdc23/Mcm10 appears to be required both for the initiation and elongation steps of DNA replication, although ts alleles do not cause an abrupt block to DNA replication in *S. pombe*, presumably reflecting leakiness of these mutations [14]. To determine the effect of Cdc23 cleavage on DNA replication, we arrested cells expressing Cdc23_S424_::Tcs in G1 by nitrogen starvation, and then released cells from the block under conditions where the TEV protease was expressed (Fig. 3A). Control cells, where the TEV protease was not induced, executed DNA replication at around 4 h after release (Fig. 3C), but in contrast expression of TEV protease prevented DNA replication (Fig. 3B), implying that the replication function of Cdc23 is inactivated by separation of the C-terminal segment of the protein. Under these conditions, expression of TEV protease in a *cdc23*^+ ^background is not deleterious (Fig. 1A, data not shown). We also investigated how cleavage of the protein in a log phase culture affected DNA replication. Because de-repression of the *nmt1 *promoter by removal of thiamine is slow, taking 12–16 h, instead we first derepressed the promoter at 25°C, when the strain is viable. After TEV protease induction, cells were shifted to 37°C to effect Cdc23_S424_::Tcs cleavage, which caused cells to arrest with slightly less than 2C DNA content (Fig. 3D). Cells are not blocked at the start of S phase but appear to arrest without completing DNA replication. Cdc23 levels are higher in a log-phase cells compared to nitrogen-starved cells [25] which may make TEV protease inactivation of Cdc23 less efficient in this experiment.

### Nuclear localization or chromatin binding of Cdc23 is not specified by the C-terminal region of the protein

**Figure 4:**
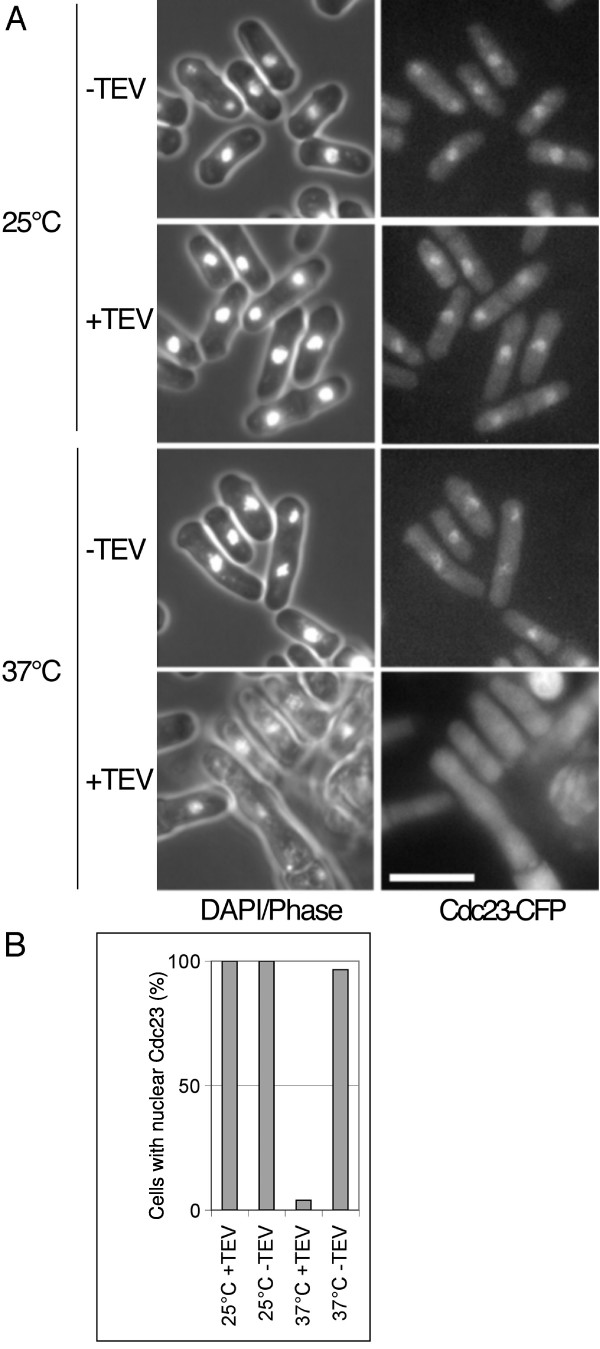
**The C-terminal fragment of Cdc23_S424_::Tcs is not localized to the nucleus after cleavage by TEV protease**. A. *cdc23_S424_::Tcs *strain (P1323) was grown in plus (-TEV) or minus thiamine (+TEV) media at 25°C for 24 h, then shifted to 37°C for 4 h before fixation with methanol and acetone. Panels show Cdc23-CFP fluorescence and DAPI staining superimposed on phase images. Bar = 10 μm. B. Quantitation of experiment shown in A. 100 cells were counted for each time point.

The previous experiments establish that Cdc23's replication function is abolished when the C-terminal 170 aa domain is cleaved off. To determine whether the C-terminal region has a function in nuclear localization, as shown in *S. cerevisiae *[32], we followed the fate of the CFP-tagged C-terminal fragment after Cdc23_S424_::Tcs cleavage. Expression of TEV protease at 25°C had no effect on the nuclear localization of Cdc23 (Fig. 4), consistent with the viability of strain under these conditions and reduced cleavage of Cdc23_S424_::Tcs at lower temperatures (Fig. 2C, data not shown). In contrast TEV protease cleavage of Cdc23_S424_::Tcs at 37°C caused the CFP signal to become dispersed all over the cell, implying that the C-terminal fragment does not contain an NLS. Since the TEV protease is targeted to the nucleus, we assume that this is the main location of Cdc23_S424_::Tcs cleavage. Thus the lack of nuclear retention of the cleaved C-terminal fragment also implies that it is incapable of binding to chromatin, although it is possible that this region contributes to Cdc23 chromatin binding in the context of the intact protein. In contrast to the C-terminal NLSs of *S. cerevisiae *Mcm10 [32], Cdc23 has a bipartite NLS near the N-terminus of the protein (aa 17-33, [27]), and taken together these results suggest that the region of the protein responsible for nuclear localization has not been conserved in evolution.

### Cleavage of Cdc23 affects the chromatin association and nuclear distribution of DNA polymerase α-primase

**Figure 5:**
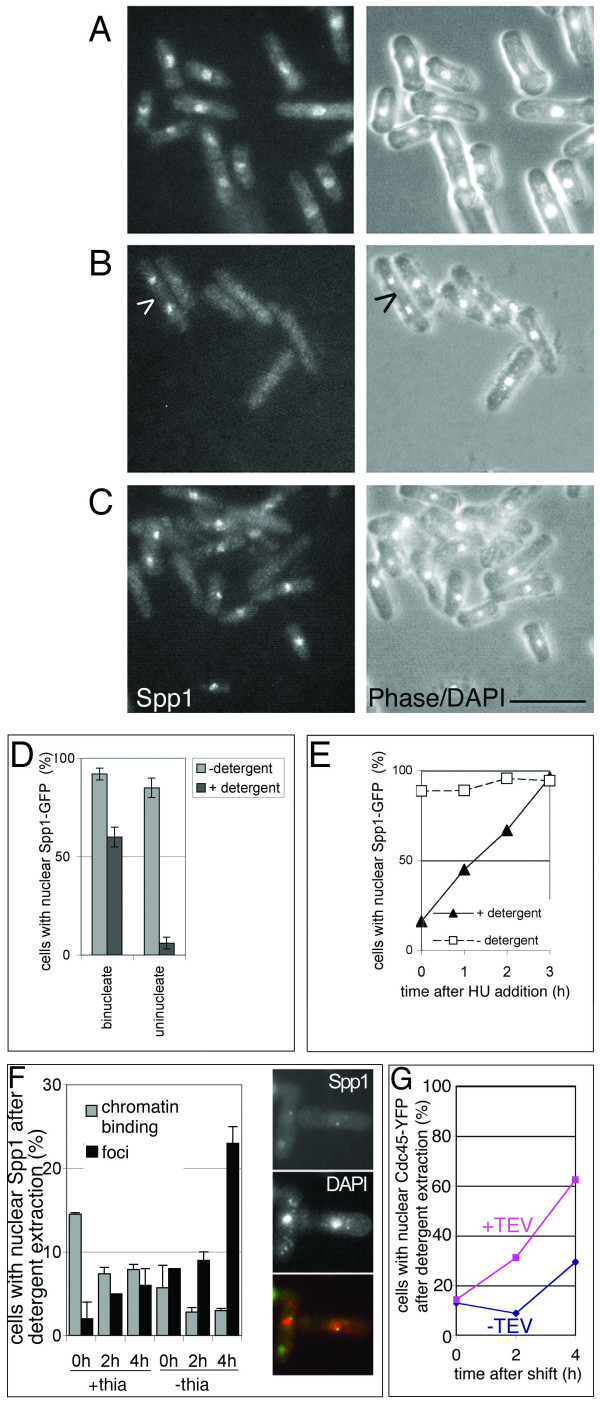
**Analysis of Spp1-GFP nuclear distribution after TEV protease cleavage of Cdc23_S424_::Tcs**. A. Localization of Spp1-GFP after direct fixation (methanol/acetone) of an asynchronous culture (strain P903). B. Localization of Spp1-GFP (strain P903) after detergent extraction of cells; >50% binuclear cells show nuclear Spp1 (arrow) C. As in (B) except that cells were grown in 12 mM HU for 2 h before analysis. Bar = 10 μm. D. Quantitation of data in (B); 100 cells were counted for each data point and 100% represents the total number of binucleate or uninucleate cells counted. E. Quantitation of data in (C); data for other time points (with and without detergent extraction) are also shown. F. Chromatin binding analysis of Spp1-GFP after inactivation of Cdc23_S424_::Tcs by TEV protease cleavage. Strain P1460 was grown either in the absence or presence of thiamine for 24 h at 25°C, then shifted to 37°C for 2, 4 h before analysis using the chromatin binding assay. Grey bars show the percentage of total cells showing Spp1-GFP fluorescence coincident with chromatin (DAPI-staining region) after detergent extraction. Black bars show the percentage of cells showing foci of Spp1-GFP fluorescence in the absence of general nuclear fluorescence (example shown in right-hand panels). At least 300 cells were counted for each data point. G. Chromatin association of Cdc45-YFP is not prevented by cleavage of Cdc23_S424_::Tcs by TEV protease. Strain P1409 was grown either in the absence or presence of thiamine for 24 h at 25°C, then shifted to 37°C for 2, 4 h before analysis using the chromatin binding assay. Data points show the percentage of total cells showing nuclear retention of Cdc45-YFP after detergent extraction. At all time points, >95% of cells show nuclear Cdc45-YFP before detergent extraction.

We used an in situ chromatin binding assay [35] to determine how inactivation of Cdc23 affects the chromatin association of pol α-primase and GINS (Sld5-Psf1-Psf2-Psf3) complexes, which are both required for the initiation and elongation steps of DNA replication. An earlier study has shown that the primase catalytic subunit, Spp1, is functional when C-terminally tagged with GFP and nuclear throughout the cell cycle [36]. However, cell cycle changes in chromatin association of Spp1-GFP are revealed by detergent extraction of permeabilized cells, using a low salt extraction buffer (Fig. 5A–D). Detergent washing removed Spp1 from uninucleate (G2) cells, but about 60% of binucleate (G1/S phase) cells retained Spp1 (Fig. 5A, B, D). Arrest of DNA replication with hydroxyurea (HU) caused Spp1 to become refractory to detergent extraction (Fig. 5C, E). Taken together, these results suggest that this assay can be used to monitor the G1/S-dependent binding of pol α primase to chromatin and is broadly consistent with western analysis of chromatin-enriched fractions [37,38].

Cleavage of the Cdc23_S424_::Tcs protein in a strain also expressing Spp1-GFP was effected by inducing the TEV protease at 25°C, and then shifting to 37°C. In a wild-type strain, this temperature shift has no effect on Spp1-GFP chromatin binding (data not shown). In contrast to the effect of HU arrest on Spp1 chromatin binding, Cdc23_S424_::Tcs cleavage caused a reduction in the proportion of cells retaining chromatin-associated Spp1 (from 14% to 3%; Fig. 5F). A reduction was also seen in thiamine-containing medium, probably reflecting leaky expression of TEV protease from the *nmt1 *promoter under repressing conditions, leading to some Cdc23 inactivation. We also observed a reduction in Spp1 chromatin binding when TEV protease was induced at 37°C in the absence of a temperature shift (data not shown). In addition to an effect on Spp1 chromatin binding, we observed a striking increase in the percentage of cells containing 1–2 bright foci of Spp1-GFP after Cdc23 inactivation (from 2% to 23%; Fig. 5F). These were generally nuclear or peri-nuclear but not necessarily co-localizing with DAPI-staining chromatin regions and may reflect formation of insoluble aggregates of Spp1 after Cdc23 inactivation. These foci were not observed during an HU-induced S phase arrest (Fig. 5C), so they appear to be a specific response to Cdc23 inactivation.

Chromatin association of pol α-primase has been reported to be dependent on Cdc45 [37,39]. Since Cdc23/Mcm10 can affect Cdc45 chromatin association [21,24,25], the effect on pol α-primase could be indirectly mediated by Cdc45. We therefore examined chromatin association of Cdc45-YFP after TEV protease inactivation of Cdc23_S424_::Tcs. In contrast to the effect on Spp1, Cdc45 chromatin association was not prevented by TEV protease cleavage of Cdc23 and in fact there was an increase in the percentage of cells with chromatin-bound Cdc45 (Fig. 5G). This is similar to what is observed in an HU arrest [25,40], presumably reflecting accumulation of cells blocked in S phase. This result suggests that the essential function of the C-terminal region of Cdc23 is not connected to Cdc45 chromatin association and may be more directly related to pol α-primase function.

**Figure 6:**
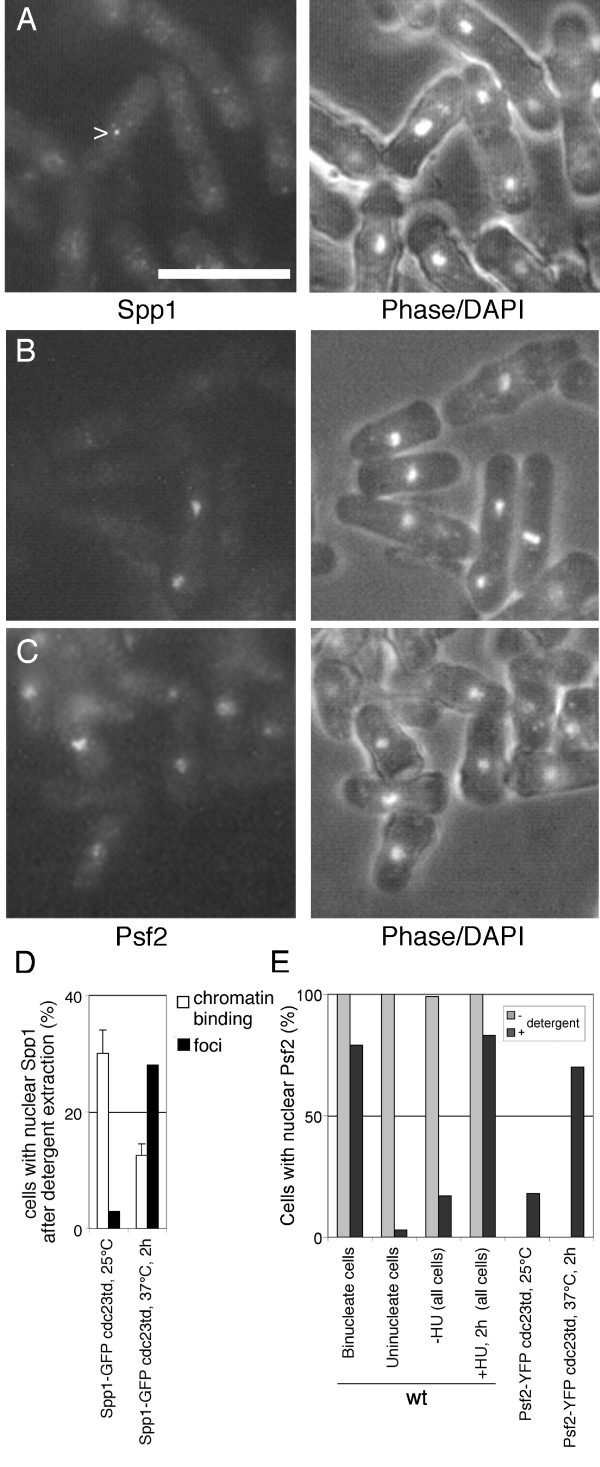
**Localization of Spp1-GFP and Psf2-YFP after inactivation of Cdc23 using a degron mutant**. A. Strain P1205 containing Spp1-GFP in the background of a degron *cdc23tstd *allele [25] was initially grown at 25°C in the absence of thiamine. 3 h before the temperature shift to 37°C, thiamine was added to repress *cdc23 *transcription, and 2 h after the shift, cells were analyzed using the chromatin binding assay (focus of Spp1-GFP shown (arrow), bar = 10 μm.) Quantitative analysis is shown in (D). B. Analysis of wild-type Psf2-YFP strain (P1411) in log phase after detergent extraction, showing retention of Psf2 in >50% binucleate (G1 or S phase) cells but not uninucleate (G2) cells. Quantitative analysis is shown in (E). C. Strain P1453, containing Psf2-YFP in the background of a degron *cdc23tstd *mutant, was treated as in (A) and analyzed by the chromatin binding assay. Cells shown had been shifted to 37°C for 2 h to inactivate Cdc23 before processing using the chromatin binding assay. Quantitative analysis is shown in (E). D. Analysis of data in experiments shown in (A); at least 200 cells were counted for each data point in each experiment. White bars show the percentage of total cells showing Spp1-GFP fluorescence coincident with chromatin (DAPI-staining region) after detergent extraction. Black bars show the percentage of cells showing foci of Spp1-GFP fluorescence in the absence of general nuclear fluorescence. 100% represents total number of cells counted for each data point. E. Analysis of Psf2-YFP in nuclei of wild-type cells, and in *a cdc23tstd *degron mutant at permissive and non-permissive temperatures, either fixed directly (-detergent), or after detergent extraction (+detergent). For the experiment with the *cdc23tstd *mutant, the percentages of all cells (i.e. binucleate and uninucleate) are shown.

To examine whether pol α-primase was also affected by a different conditional allele of Cdc23, we also constructed a strain expressing Spp1-GFP in the background of a degron *cdc23tstd *allele (this allele combines a ts degron [41] with the ts *cdc23-1E2 *allele, [25]). In contrast to the effect of HU, a reduction in Spp1 chromatin binding occurred on S phase arrest by Cdc23 inactivation (Fig. 6A, D). As with the experiment using Cdc23_S424_::Tcs cleavage, degron inactivation of Cdc23 also caused an increase in the proportion of cells showing foci of Spp1-GFP (Fig. 6D). Taken together these observations suggest that Cdc23 is needed for the normal chromatin association and subnuclear distribution of pol α-primase.

The GINS complex has been shown to be loaded onto chromatin at initiation in *S. cerevisiae *and *Xenopus *[42-45] and we examined whether this complex is also affected by disruption of Cdc23 function. We first determined whether GINS shows cell cycle dependent changes in detergent extractability. A strain where the Psf2 subunit of GINS is C-terminally tagged with YFP is viable and shows nuclear localization throughout the cell cycle (Fig. 6E). After detergent extraction, Psf2 is only retained in binucleate cells, and is resistant to detergent extraction in HU-arrested cells (Fig. 6B, E), suggesting that the protein is chromatin associated in S phase similar to results obtained with Spp1. After shifting the *cdc23tstd *strain to the non-permissive temperature, Psf2 becomes resistant to detergent extraction, similar to what is observed in an HU arrest (Fig. 6C, E). Similar results were obtained after TEV protease cleavage of Cdc23 (data not shown). This suggests Cdc23 is not required to maintain Psf2 chromatin association and execution of the Cdc23 function is needed for displacement of Psf2 in the course of S phase.

## Discussion

In this paper we demonstrate that in vivo cleavage by TEV protease can be used to inactivate a modified version of the Cdc23 replication factor, engineered to contain a cleavage site for the protease. We infer that the C-terminal 170 aa region is essential for the DNA replication function of Cdc23. Since N- and C-terminal fragments appear to be stable after cleavage, the C-terminal region must require covalent linkage to the rest of the protein for its function. Although no clear sequence motifs have been identified in the C-terminal region, this part of the protein could have a discrete function that requires linkage to the N-terminal domain. Alternatively, interaction between the N and C-terminal regions could simply be necessary for the function of the protein in a manner which is affected by TEV protease cleavage. This function does not appear to involve nuclear localization or chromatin binding, since nuclear localization of the C-terminal fragment is lost after Cdc23 cleavage (Fig. 4).

We also show that inactivation of Cdc23, either by proteolytic cleavage or using a ts degron allele, affects the chromatin association and nuclear distribution of Spp1 primase subunit (Figs. 5, 6), suggesting that an essential role of Cdc23 is related to recruitment of pol α-primase to chromatin. Cdc45 has also been implicated in binding of pol α-primase to chromatin [37,39], but in this context it may not be relevant. Although complete inactivation of Cdc23/Mcm10 can block chromatin association of Cdc45 [21,24,25], TEV protease cleavage of Cdc23 does not prevent Cdc45 chromatin binding. One interpretation of these results is that Mcm10/Cdc23 has two functions, one related to pol α primase function, perhaps related to the C-terminal domain cleaved off by TEV protease, and the other related to Cdc45 chromatin binding.

These results are consistent with in vitro effects of *S. pombe *Cdc23 on pol α-primase activity [27], and evidence that *S. cerevisiae *Mcm10 promotes chromatin binding of the large primase subunit (Pri2) independently of effects on Cdc45, based on histone cross-linking experiments [20]. The observations we have made for Spp1-GFP may also apply to the three other subunits of pol α-primase (p49/Spp2, p70/B-subunit, p180/Pol1). However, given the requirement for *S. cerevisiae *Mcm10 for stability of the p180 polymerase catalytic subunit [20] and the constitutive chromatin binding of at least a fraction of *S. pombe *p70 [38], further work will be needed to determine the fate of other pol α primase subunits on Cdc23 inactivation.

In contrast to effects on Spp1, chromatin association of the Psf2 subunit of GINS is seen in cells arrested in S phase by using a degron allele of *cdc23 *(Fig. 6), similar to the result obtained by an HU arrest of DNA replication. This suggests that maintenance of GINS chromatin association is independent of Cdc23 function and that Psf2 displacement requires Cdc23 function in the elongation step of replication. At first glance, this result is inconsistent with previous work linking Cdc45 with GINS function. Specifically, chromatin association of Sld5 in Xenopus extracts is blocked by Cdc45 depletion [43], and in *S. cerevisiae*, the Sld3 partner of Cdc45 is essential for Psf1 origin association [42]. However, degradation of Cdc23 using the *cdc23tstd *allele used in our experiments is inefficient in cycling cells [25] and Cdc45 chromatin binding is not blocked under conditions of the Psf2 experiment shown in Fig. 6 (see Additional file: 1).

In this and a related study [13] we have demonstrated the utility of TEV protease for analysis of protein function in fission yeast. TEV protease can be used to determine the role of specific domains of the protein and this method can be used for topological analysis of protein function by restricting TEV protease expression to specific compartments. For this approach to be useful, the target protein must tolerate the insertion of a Tcs, which was an issue with our related study on Pol3 function [13] and the Tcs must be accessible to TEV protease in the folded protein. In this study, cleavage of Cdc23_S424_::Tcs was more efficient at higher temperatures, possibly reflecting a conformational change in Cdc23 which makes the Tcs more accessible to the protease. Alternatively, the interaction of Cdc23 with other factors might be changed by temperature in a manner which affects access to the Tcs.

In the approach used here, the Tcs generates a C-terminal fragment with serine as the N-terminal amino acid, which is predicted to generate a stable protein. However, TEV protease also cleaves sequences such as ENLYFQ^∨^R [5,46], which generates a C-terminal fragment with a destabilizing N-terminal amino acid according to the N-end rule. Such a module can be inserted at the N-terminus of the target protein, which may be more tolerated and accessible than insertion of the Tcs at internal sites. We are currently investigating whether this strategy provides a simpler approach to effect rapid inactivation of target proteins.

## Conclusion

We show that TEV protease can be used in *S. pombe *to inactivate target proteins engineered to contain a protease cleavage site. Using this approach we show that the C-terminal domain of the Cdc23/Mcm10 replication factor, previously with no attributed functions, is essential for DNA replication. Inactivation of Cdc23/Mcm10 affects the chromatin binding and nuclear distribution of pol α-primase, suggesting it may help to attach the polymerase to the replication fork. In contrast, Cdc23/Mcm10 inactivation does not prevent maintenance of chromatin binding of the GINS elongation factor, as judged by analysis of the Psf2 subunit.

## Methods

### Fission yeast strains

Fission yeast strains used were constructed by standard genetic methods and are shown in Table 1. Strains were grown in rich medium (YE3S) or minimal medium (EMM). Repression of transcription from the *nmt1 *promoter was achieved by addition of thiamine (15 μg/ml) to EMM.

### Construction of a plasmid expressing TEV protease under *nmt1 *regulation(pREP3X-TEV-NLS)

A fragment encoding NLS-myc9-TEV-NLS-NLS was amplified from plasmid YIp204 (GAL-NLS-myc9-TEV proteinase-NLS-NLS, [9]) using oligonucleotide primers 5'XhoITEV204 (TTTCTCGAGAGAAAAAACCCCGGATCTATGCCAA) and 3'SmaITEV204 (AAACCCGGGGCCAAGCTTGCATGCCTGCAGTTAATC) and cloned into XhoI and SmaI sites of the plasmid pREP3X [47], to generate the pREP3X-TEV-NLS plasmid.

### Construction of *mcm7 *and *cdc23 *alleles containing TEV cleavage sites

To construct *mcm7-GFP*_*N760*_*::Tcs*, we first constructed a plasmid pSMUG2+mcm7 by subcloning the ApaI-SmaI fragment from pSMUC2+mcm7 [25] into pSMUG2+ [48]. The sense oligonucleotide 5'EspI-TEV-KasI-F (CCGGGTTACTTCTTTAGAAGTTGGTCGTCGCTTTTCTCCTGATGAAAATTTGTACTTCCAATCTGGCGC) and anti-sense oligonucleotide 5'KasI-TEV-EspI-R (CCGGGCGCCAGATTGGAAGTACAAATTTTCATCAGGAGAAAAGCGACGACCAACTTCTAAAGAAGTAAC) were annealed and cloned into the XmaI site of pSMUG2+mcm7. This was used to tag the endogenous *mcm7*^+ ^gene by cutting with MluI and transforming into strain P138 to generate P1288. The sequence inserted after the terminal amino acid of Mcm7, before the GA linker-GFP module is RVTSLEVGRRFSPDENLYFQS (underlined sequence is the Tcs).

Two rounds of PCR were performed to construct the *cdc23*_*S424*_*::Tcs *allele. Initially, two PCR reactions were set up, using plasmid pSMUC2+Cdc23 as DNA template, using the primers: (i) 5'ApaIMCM10 (AAAGGGCCCGAACCA AGAAAGGAAGAGGAGCGATAA) and 3'MCM10tevAclI1010 (CCCGTAGAG AGGTTATTCGACTGGAAGTACAACGTTTCGGAAGCGCAACATCGC); (ii) 5' MCM10tevAclI1010 (GCGATGTTGCGCTTCCGAAACGTTGTACTTCCAGTC GAATAACCTCTCTACGGG) and 3' ATGCYfp-screen (ACAGCTCCTCGCCCTTGCTCACCAT). Products from the two PCR reactions were mixed and boiled and used for a secondary PCR reaction using primers 5'ApaIMCM10 and 3' ATGCYfp-screen. The PCR product was cloned into ApaI and HindIII sites of plasmid pSMUC2-Cdc23 [25]. This was used to tag endogenous *cdc23*^+ ^gene by cutting with SpeI and transforming into strain P138 to generate P1322. All constructs were verified by sequencing.

### Construction of strains expressing fluorescently-tagged Spp1 and Psf2

The catalytic subunit of DNA primase (Spp1) was tagged with GFP using the oligos p48F1 (TGTACTTTAAATCGTTCAGTAGCCAGCTTTTTAAAGAAACAGTAGGAAATAAAAGAAAACATGAGAATTTGGAATTTCGGATCCCCGGGT) and p48R1 (GATTTAACGGAAAACAATATGCTCCACGTAAATAGAAGTCAGAGCTTCCATCCTTTCATGAGTAAATGTATGCCCAAATGAATTCGAGCTCGTTTAAACT) using pFA6a-GFP-kanMX6 as template [49] to generate a PCR product that was used to tag the endogenous Spp1 gene by transforming strain P138. Psf2 was tagged with YFP using the oligos 5'ApaI-Psf2 (CTTGGGCCCATACGAGATGATGAATTGGAAAACG) and 3'XhoIPsf2 (TTTCTCGAGTTCTTCTTGGGAAACTTGAACAAT) which was cloned into pSMUY2+ [25]; cleavage of this plasmid with EcoRV was used to tag the endogenous *psf2*^+ ^gene in strain P138 with YFP, generating strain P1411. All constructs were verified by sequencing. Strains containing Spp1-GFP or Psf2-YFP showed tagged proteins with the expected molecular weights when analyzed by western blotting, using anti-GFP antibody (data not shown).

### Chromatin binding assay

Chromatin binding assay and image analysis was carried out as previously described for Psf2 [35,50]. For analysis of Spp1, a low-salt extraction buffer was used with the following composition: 20 mM Pipes-KOH pH 6.8, 0.4 M sorbitol, 10 mM KAc, 0.5 mM spermidine, 0.15 mM spermine, 1 mM EDTA. Extraction with higher salt buffers results in loss of Spp1 from S phase cells (data not shown). Data shown are the averages of at least two experiments. Nuclear retention of Spp1-GFP and Psf2-YFP after detergent extraction was abolished after digestion of chromatin with micrococcal nuclease (data not shown). Chromatin binding assays were performed at least twice and error bars show the statistical range. At least 100 cell were counted for each data point.

### Flow cytometry

Flow cytometry was carried out using sytox green (1 μm) as previously described [25].

### Western blotting

Protein extracts for Western blotting were made by trichloroacetic acid extraction as previously described [51]. For western blot analysis, antibodies against full-length Cdc23 (from S. Aves [25]), GFP (3E1 monoclonal), TEV protease (from M. Ehrmann), and α-tubulin (Sigma T5168) were used. Detection was performed using the chemiluminescence procedure.

## Authors' contributions

XY carried out most of the experiments. JG devised conditions for the Spp1 chromatin binding assay. KL constructed strains. HY contributed to the experiments shown in Figs. 5, 6. SK designed the experiments and wrote the paper.

## Supplementary Material

Additional File 1 **Chromatin binding analysis of Cdc45-YFP in a degron *cdc23td *strain, in asynchronous culture**. A. Scheme of experiment shown in (B). Cultures of P1083 (*cdc45-YFP*) and P1100 (cdc45-YFP *cdc23tstd*) were grown at 25°C to log phase. HU (12 mM) was added and the cultures were split; half the cells were shifted to 37°C. Chromatin binding analysis was carried after on the -HU 25°C cells, and on cells from the +HU cultures after 3 h. B. Analysis of cells with nuclear Cdc45 either with or without detergent extraction. The control strain shows an increase in chromatin-associated Cdc45 (i.e. nuclear Cdc45 after detergent extraction) during the HU arrest either at 25°C or 37°C as previously reported [25], as displacement of Cdc45 from chromatin at the end of S phase is prevented by the S phase arrest. In the *cdc23tstd *strain, a similar result is shown, indicating that the degron allele does not affect Cdc45-YFP chromatin association. This may reflect inefficient inactivation of Cdc23 under these conditions. In contrast to this result, Cdc45 chromatin association is affected when cdc23 is inactivated following G1 arrest by nitrogen starvation [25]. Click here for file

## Figures and Tables

**Table 1:** *S. pombe *strains used

		reference
P138	*ade6-M210 leu1-32 ura4-D18 h*^-^	this work
P903	*spp1-GFP::kan*^*r *^*ade6-M120 leu1-32 ura4-D18 h*^-^	[25]
P1083	*cdc45(sna41)-YFP::ura4*^+^*ade6-M210 leu1-32 ura4-D18 h*^-^	[25]
P1100	*cdc45(sna41)-YFP::ura4*^+^nmt1*cdc23-1E2-td::kan^*r *^*h*^-^	this work
P1171	*mcm7-GFP*_*N*760_*::Tcs ::ura4*^+^*ade6-M210 leu1-32 ura4-D18 h*^- ^*[pREP3X-TEV-NLS(LEU2)]*	this work
P1205	*spp1-GFP::kan*^*r *^nmt1*-cdc23-1E2-td::kan^*r *^*leu1-32*	this work
P1288	*mcm7-GFP*_*N*760_*::Tcs ::ura4*^+^*ade6-M210 leu1-32 ura4-D18 h*^-^	this work
P1292	*ade6-M210 leu1-32 ura4-D18 h*^- ^*[pREP3X-TEV-NLS(LEU2)]*	this work
P1322	*cdc23-CFP*_*S*424_*::Tcs::ura4*^+^*ade6-M210 leu1-32 ura4-D18 h*^-^	this work
P1323-1 P1323-2	*cdc23-CFP*_*S*424_*::Tcs::ura4*^+^*ade6-M210 leu1-32 ura4-D18 h*^- ^*[pREP3X-TEV-NLS(LEU2)]*	this work
P1376	*cdc23-CFP*_*S*424_*::Tcs::ura4*^+^*leu1-32 ura4-D18 h*^- ^*[pREP3X-TEV-NLS(LEU2)]*	this work
P1409	*cdc45(sna41)-YFP::ura4*^+^*cdc23-CFP*_*S*424_*::Tcs::ura4*^+^*leu1-32 ura4-D18 ade6 [pREP3X-TEV-NLS(LEU2)]*	this work
P1411	*psf2-YFP::kan*^*r*^*ade6-M210 leu1-32 ura4-D18 h*^-^	this work
P1453	*psf2-YFP::kan*^*r *^nmt1*-cdc23-1E2-td::kan^*r*^	this work
P1460	*spp1-GFP::kan*^*r *^*cdc23-CFP*_*S*424_*::Tcs::ura4*^+^*leu1-32 ura4-D18 ade6 [pREP3X-TEV-NLS(LEU2)]*	this work

*medium-strength *nmt1 *promoter
