# Supplementary material for: Nuclear distribution and chromatin association of DNA polymerase α-primase is affected by TEV protease cleavage of Cdc23 (Mcm10) in fission yeast
Source: BMC Mol Biol. 2005 Jun 7;6:13. doi: 10.1186/1471-2199-6-13 (PMC1182370; doi:10.1186/1471-2199-6-13)
Supplement: Additional File 1 — Chromatin binding analysis of Cdc45-YFP in a degron cdc23td strain, in asynchronous culture. A. Scheme of experiment shown in (B). Cultures of P1083 (cdc45-YFP) and P1100 (cdc45-YFP cdc23tstd) were grown at 25°C to log phase. HU (12 mM) was added and the cultures were split; half the cells were shifted to 37°C. Chromatin binding analysis was carried after on the -HU 25°C cells, and on cells from the +HU cultures after 3 h. B. Analysis of cells with nuclear Cdc45 either with or without detergent extraction. The control strain shows an increase in chromatin-associated Cdc45 (i.e. nuclear Cdc45 after detergent extraction) during the HU arrest either at 25°C or 37°C as previously reported [25], as displacement of Cdc45 from chromatin at the end of S phase is prevented by the S phase arrest. In the cdc23tstd strain, a similar result is shown, indicating that the degron allele does not affect Cdc45-YFP chromatin association. This may reflect inefficient inactivation of Cdc23 under these conditions. In contrast to this result, Cdc45 chromatin association is affected when cdc23 is inactivated following G1 arrest by nitrogen starvation [25]. [file 1471-2199-6-13-S1.pdf]

A

B

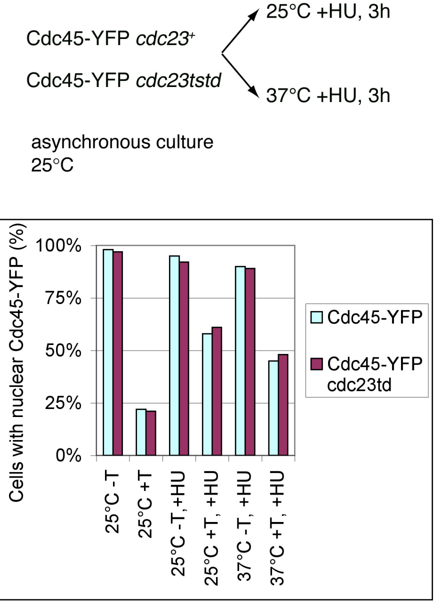

Chromatin binding analysis of Cdc45-YFP in a degron cdc23td strain, in asynchronous culture.

A. Scheme of experiment shown in (B). Cultures of P1083 (cdc45-YFP) and P1100 (cdc45-YFP cdc23tstd) were grown at 25°C to log phase. HU (12 mM) was added and the cultures were split; half the cells were shifted to 37°C. Chromatin binding analysis was carried after on the –HU 25°C cells, and on cells from the +HU cultures after 3h. B. Analysis of cells with nuclear Cdc45 either with or without detergent extraction. The control strain shows an increase in chromatin-associated Cdc45 (i.e. nuclear Cdc45 after detergent extraction) during the HU arrest either at 25°C or 37°C as previously reported [25], as displacement of Cdc45 from chromatin at the end of S phase is prevented by the S phase arrest. In the cdc23tstd strain, a similar result is shown, indicating that the degron allele does not affect Cdc45-YFP chromatin association. This may reflect inefficient inactivation of Cdc23 under these conditions. In contrast to this result, Cdc45 chromatin association is affected when cdc23 is inactivated following G1 arrest by nitrogen starvation [25].
